# Prior-aligned frequency-domain explanations for heart sound classification: a scale-consistent attribution approach

**DOI:** 10.3389/frai.2026.1780496

**Published:** 2026-05-18

**Authors:** Qiyang Sun, Yupei Li, Aydin Javadov, Xiaoliang Wu, Björn W. Schuller

**Affiliations:** 1 Group on Language Audio & Music, Department of Computing, Imperial College London, London, United Kingdom; 2 Chair of Health Informatics (CHI), TUM University Hospital, Munich, Germany; 3 Mobiliar Lab for Analytics, Swiss Federal Institute of Technology, Zurich, Switzerland; 4 School of Electronics and Computer Science, University of Southampton, Southampton, United Kingdom; 5 Munich Data Science Institute (MDSI), Munich, Germany; 6 Munich Center for Machine Learning (MCML), Munich, Germany

**Keywords:** attribution regularization, deep learning, explainability, explainable artificial intelligence (XAI), heart murmurs, heart sound classification, phonocardiogram (PCG), spectral analysis

## Abstract

**Objective:**

Deep learning models for phonocardiogram (PCG) classification often achieve high accuracy but lack transparency in relation to clinically meaningful features. Existing explainable artificial intelligence (XAI) methods rarely ensure alignment between model attributions and cardiac physiology.

**Methods:**

We propose Scale-Consistent Attribution (SCA), a training-time regularization method that incorporates domain knowledge into model learning. SCA aligns spectral attention with a soft clinical prior, distinguishing low-frequency heart sounds from high-frequency murmurs. The method is evaluated on the PhysioNet 2016 and CirCor DigiScope datasets.

**Results:**

SCA remarkably improves the physiological plausibility of explanations. Specifically, it reduces the divergence between model attribution and clinical priors by an order of magnitude. Crucially, the model maintains competitive classification accuracy. Ablation studies with incorrect priors confirm that these benefits stem from accurate clinical medical knowledge. Qualitative and quantitive analysis further reveals that SCA shifts model focus towards clinically relevant frequency regions.

**Conclusion:**

SCA offers a robust framework for trustworthy and explainable PCG classifiers, improving the clinical plausibility of explanations while maintaining strong predictive performance.

## 1 Introduction

Cardiovascular diseases (CVDs) pose severe threats to public health. According to the World Health Organization (WHO), approximately 19.8 million people died from CVDs in 2022, accounting for 32% of all global deaths (Mensah et al., 2023). While advanced diagnostic modalities like echocardiography and cardiac magnetic resonance imaging (cardiac MRI) (Sun, 2025; Lee and Park, 2023) provide definitive assessments, they are often limited by high costs, the need for specialized operators, and low accessibility in primary care settings. Furthermore, invasive procedures such as angiography (Sanfilippo et al., 2023) can increase patient suffering and procedural risks. Consequently, these barriers often lead to delayed diagnoses and missed opportunities for early intervention. In this context, Phonocardiogram (PCG) analysis (Zhu et al., 2024) offers a non-invasive, cost-effective solution for large-scale screening, serving as a crucial bridge in resource-constrained environments.

Rapid advancements in AI have remarkably unlocked the diagnostic potential of PCG signals. Deep learning (DL) models, such as Convolutional Neural Networks (CNNs) and Transformers, possess the ability to automatically learn complex hierarchical representations (Zhao et al., 2024). These models extract features directly from raw waveforms or time-frequency spectrograms. Recent studies utilizing large-scale public benchmarks confirm the clinical promise of these data-driven approaches. For instance, on the PhysioNet 2016 benchmark (Clifford et al., 2016), Kalimuthu and Hemanth (2025) proposed a modified ResNet integrated with a Convolutional Block Attention Module (CBAM). They report a classification accuracy of 95.21% in binary classification. This demonstrates the capacity of deep features to capture subtle pathological changes in controlled environments. Even in challenging real-world scenarios characterized by background noise, such as the CirCor DigiScope dataset (Oliveira et al., 2021), performance remains robust. Zhang et al. (2024) introduce a parallel-attentive model combined with uncertainty estimation. This method achieved an F1-score of 65.1% in the murmur detection challenge. Additionally, Sabu et al. (2025) propose a deep learning-based framework that employs Mel-Frequency Cepstral Coefficients (MFCCs) and a Deep Neural Network (DNN) to classify heart sounds. By extracting key spectral features, their model achieved an accuracy of 63.04%, showcasing effectiveness in uncontrolled environments.

DL achieves impressive statistical performance on various PCG benchmarks. However, its “black-box” nature remains an unavoidable barrier to clinical adoption and trust (Sun et al., 2025; Li et al., 2025; Javadov et al., 2025). Current automated PCG analysis methods often rely on *post-hoc* explainability or the visualization of attention weights. For instance, Ren et al. (2022) used deep attention networks to locate key temporal frames in heart sounds. Özcan (2024) applied Grad-CAM to visualize murmur regions within transfer learning models. Similarly, Sharma et al. (2025) combined SpectroNet-LSTM with *post-hoc* attribution to identify suspicious heartbeat segments. These methods face two critical limitations. First, the resulting explanations often lack clear clinical semantics. Existing heatmaps are frequently noisy and difficult to reproduce. As shown in current studies, they focus on when an anomaly occurs rather than whether the model relies on specific spectral structures. Second, these approaches represent passive dissections of trained models rather than active constraints on the learning process. They observe attention patterns *post-hoc* but cannot guarantee alignment with established cardiac physiology. PCG research currently lacks a systematic mechanism to encode clinical priors directly into the training objective. Furthermore, there are no quantifiable metrics to optimize the alignment between model explanations and frequency-domain knowledge. Without such constraints, models may capture background noise or equipment frequency shifts instead of valid pathological features.

To address these challenges, we propose Scale-Consistent Attribution (SCA), a training-time regularization method. SCA does not alter existing model architectures. Instead, it operates on log-Mel spectrograms. Based on cardiac physiology (Arnott et al., 1984; Atanasov and Ning, 2008), we partition the spectrum into four frequency bands with distinct clinical semantics. We then construct class-related frequency prior distributions. Through Kullback–Leibler (KL) divergence, we explicitly align the model's gradient attribution distribution with these priors. Intuitively, this mechanism encourages the model to rely on low-frequency components when identifying normal heart sounds. For pathological anomalies, the model focuses on mid-to-high frequency energy. This approach avoids over-reliance on high-frequency noise or equipment artifacts.

We conduct systematic experiments on the PhysioNet 2016 and CirCor DigiScope benchmarks. Results show that SCA maintains or slightly improves classification accuracy compared to strong baselines. Crucially, SCA reduces the KL divergence between model attributions and clinical frequency priors by approximately one order of magnitude. The method remains robust under various ablation conditions, including incorrect priors and uniform priors. These findings suggest that the model learns decision patterns aligned with cardiac physiology. This enhances both explainability and trustworthiness in clinical settings.

The main contributions of this work are summarized below:

- We propose the SCA regularization framework. This introduces a model-agnostic, training-time constraint. It integrates frequency-domain clinical knowledge directly into the deep learning optimisation process.
- We establish a physiologically aware attribution alignment mechanism. Using spectral partitioning and divergence, we create quantitative metrics. These metrics explicitly guide model decision logic toward cardiac physiological principles.
- We perform multi-dimensional quantitative evaluations. On two major PCG benchmarks, we verify that SCA improves explanation quality and clinical consistency. It achieves this without sacrificing diagnostic precision.
- We conduct rigorous ablation analyses. Comparisons with “incorrect priors” and feature-space regularization confirm the source of performance gains. Visual and auditory case studies further prove that these improvements result from precise domain knowledge encoding.

## 2 Materials and methods

### 2.1 Heart sound classification

Cardiac auscultation remains one of the most fundamental and cost-effective non-invasive modalities for cardiovascular screening (Montinari and Minelli, 2019). Clinicians typically conduct systematic assessments across the four standard auscultatory areas on the chest wall: the Aortic Valve (AV), Pulmonary Valve (PV), Tricuspid Valve (TV), and Mitral Valve (MV) regions. This process involves the meticulous discrimination of the first (S1) and second (S2) heart sounds, alongside subtle acoustic variations occurring during systole and diastole (Riley, 2024). Under normal physiological conditions, S1 and S2 correspond to the closure events of the atrioventricular and semilunar valves, respectively. Their acoustic energy is predominantly concentrated within the low-frequency spectrum, characterized by a regular rhythm and distinct tonal clarity. Conversely, haemodynamic turbulence resulting from valvular pathologies or structural defects generates high-frequency cardiac murmurs (Ahmad et al., 2025). Furthermore, additional pathological acoustic features, such as gallop rhythms (S3/S4), splitting, or friction rubs, may also be present. Consequently, clinical diagnosis relies on the comprehensive analysis of the timing, frequency, intensity, and morphology of these anomalous sounds, as well as their spatial distribution across different auscultatory sites, to infer the risk of underlying structural heart disease.

In this study, we define the task as Clinical Outcome Prediction. Given a resampled and normalized single-channel PCG signal *x*∈ℝ^*T*^ (transformed into a log-Mel spectrogram), we train a deep neural network, *f*_θ_, to predict the patient's clinical status *y*∈{0, 1}. Here, *y* = 0 denotes a ‘Normal' state, while *y* = 1 corresponds to the presence of a ‘clinically relevant abnormality', as diagnosed by experienced cardiologists.

### 2.2 Datasets and preprocessing

#### 2.2.1 The PhysioNet 2016 dataset

The PhysioNet/Computing in Cardiology Challenge 2016 dataset (Clifford et al., 2016) contains 3,240 heart sound recordings. These single-lead PCG records were primarily collected in outpatient and community screening environments. Each recording corresponds to a single auscultation location. Clinical experts labeled each recording as either “Normal” or “Abnormal.” However, the official test set is not publicly available, and the provided validation set is merely a subset of the training data. To prevent data leakage, we employed a specific splitting strategy. We randomly divided the publicly available training set into training and validation subsets using a 9:1 ratio. A fixed random seed 2025 was used for the PyTorch pseudo-random number generator to ensure reproducibility. This resulted in 2,916 training recordings and 324 validation recordings.

#### 2.2.2 The CirCor DigiScope dataset

The CirCor DigiScope dataset (Oliveira et al., 2021) comprises 5,272 PCG recordings from 1,568 patients. This dataset focuses primarily on the pediatric and youth population. For each subject, recordings were collected from the four standard auscultation locations. The recording durations range from 4.8 to 80.4 seconds, totalling over 33.5 h of audio data. Comprehensive metadata accompanies each subject, including demographics and detailed murmur annotations. We obtained the official data splits (training, validation, and testing) directly from the original authors. This ensures strict subject-level independence. The final dataset distribution consists of 3,163 training, 486 validation, and 1,623 testing recordings. The detailed distribution of these two datasets is presented in Table 1.

**Table 1:** Distribution of recordings across the PhysioNet 2016 and CirCor DigiScope datasets.

Dataset	Training	Validation	Testing	Total
PhysioNet 2016	2,916	324	-	3,240
CirCor DigiScope	3,163	486	1,623	5,272

#### 2.2.3 Data pre-processing

To unify the feature representation and retain critical physiological acoustic information, we perform a standardized pre-processing pipeline on all raw heart sound recordings. All audio signals are resampled to 2,000 Hz. This sampling rate sufficiently covers the fundamental heart sound components and pathological murmurs while effectively filtering high-frequency ambient noise. To ensure data integrity, we adopted a dynamic zero-padding strategy, which dynamically fills all samples in the same batch with the length of the longest sample in that batch. Signals are subsequently normalized by their absolute maximum amplitude to ensure consistent scales across all samples. We then transform the time-domain signals into log-Mel spectrograms. The feature extraction parameters include a 50 ms window length, a 25 ms hop length, and a 256-point Fast Fourier Transform. We apply 128 Mel filter banks, that is, *F* = 128. The resulting feature tensor has a shape of [1, *T, F*]. The frequency range of the spectrogram is restricted to between 25 Hz and 1 kHz to focus on diagnostic cardiac information.

### 2.3 Clinical priors and scale-consistent attribution

#### 2.3.1 Spectral partitioning

As discussed in Section 2.1, CVDs auscultation is governed by distinct physiological acoustic characteristics. Clinical literature indicates that normal heart sounds (S1 and S2) concentrate their energy primarily in the lower frequency spectrum, typically between 20 Hz and 150 Hz (Arnott et al., 1984). Conversely, pathological murmurs typically manifest higher frequency components, generally ranging from 200 Hz to approximately 600 Hz (Atanasov and Ning, 2008). The highest frequency bands (typically exceeding 600 Hz) are often dominated by respiratory sounds, ambient noise, and equipment artifacts. According to these characteristics, we partition the 128-dimensional log-Mel spectrogram (covering a physical bandwidth of 25–1,000 Hz) into four clinically semantic frequency bands. The non-linear Mel scale naturally facilitates a rational stratification from dense low-frequency resolution to sparse high-frequency coverage. By converting the Mel indices to approximate physical frequencies, we define these four continuous 32-dimensional slices as follows:

- **Band 1 (Indices 0:32, approx. 25–175 Hz)—Fundamental Zone:** This band focuses on the primary energy of S1 and S2. It represents the core component of normal heart sounds.
- **Band 2 (Indices 32:64, approx. 175–390 Hz)—Transitional Zone:** This band covers low-to-mid frequency signals. It typically captures early systolic or diastolic murmur components.
- **Band 3 (Indices 64:96, approx. 390–660 Hz)—Pathological Zone:** This band captures the critical mid-to-high frequency features of pathological murmurs up to 600 Hz. It is critical for diagnosing abnormalities.
- **Band 4 (Indices 96:128, approx. 660–1,000 Hz)—Noise Zone:** This band consists mostly of environmental noise and artifacts beyond the typical cardiac acoustic range. It carries negligible diagnostic information.

The four-band partitioning mathematically bridges the Mel-scale feature extraction with established cardiac physiology. It balances clinical semantic clarity with computational efficiency, offering more robust and explainable alignment constraints than finer-grained or continuous modeling.

#### 2.3.2 Clinical priors

Based on this partitioning, we construct class-dependent Frequency Priors (*P*_prior_). For a given clinical status *y* ∈ {0, 1}, the prior is defined as a 4-dimensional probability vector **p**^(*y*)^ ∈ ℝ^4^, such that $\overset{4}{\underset{i=1}{\sum }}p_{i}^{\left(y\right)}=1$.

For Normal samples (*y* = 0), we define the prior as:


$$\begin{array}{l} {\text{p}}^{\left(0\right)}=\left[0.7,0.2,0.1,0.0\right], \end{array}$$ (1)

This distribution enforces attribution concentration within Band 1 (Fundamental Zone). It permits minor energy in Bands 2, 3, while suppressing Band 4. This aligns with the physiological dominance of low-frequency components in healthy heart sounds.

For Abnormal samples (*y* = 1), we define the prior as:


$$\begin{array}{l} {\text{p}}^{\left(1\right)}=\left[0.15,0.35,0.5,0.0\right], \end{array}$$ (2)

This distribution reflects the significant energy contribution of pathological murmurs in the mid-to-high frequencies (Bands 2, 3). It guides the model to focus on potential lesions while reducing reliance on the fundamental wave and high-frequency noise.

It is important to note that these priors do not aim to reconstruct the exact spectral morphology of specific diseases. Instead, they serve as a differentiable and adjustable approximation of the clinical consensus: “normal sounds are low-frequency; murmurs are mid-to-high frequency; high frequencies are noise.” In addition, values in the prior vector *p*^(*y*)^ denote relative ordinal priorities based on clinical consensus, not absolute physical energy ratios. To ensure methodological consistency, we apply the same 4-band partitioning and prior weights across all datasets in this study.

#### 2.3.3 Scale-consistent attribution

To explicitly integrate clinical prior knowledge into the deep neural network optimisation process, we propose the Scale-Consistent Attribution (SCA) method. SCA calculates the model's reliance on specific frequency bands in real-time. It then uses KL divergence to constrain this reliance to match physiological principles. The process consists of three main steps:

##### 2.3.3.1 Saliency computation via gradient × input

First, we measure which frequency bands the model actually relies on during training. We compute the saliency of the input spectrogram for each mini-batch. Let **X** ∈ ℝ^*B*×1 × *T*×*F*^ be a batch of log-Mel spectrogram inputs (where *B* is the batch size). Let $\text{z}=f_{\theta }\left(\text{X}\right)\in {\mathbb{R}}^{B\times 2}$ be the model's un-normalized output logits.

We extract the scalar score *s* corresponding to the true class *y*_*i*_ for each sample:


$$\begin{array}{l} s=\overset{B}{\underset{i=1}{\sum }}z_{i,y_{i}}. \end{array}$$ (3)

Next, we compute the gradient of *s* with respect to the input **X**, denoted as $\text{G}=\frac{\partial s}{\partial \text{X}}$. To obtain a physically meaningful attribution map, we employ the Gradient × Input method (Shrikumar et al., 2017). The saliency tensor **A** is defined as:


$$\begin{array}{l} \text{A}=|\text{G}⊙\text{X}|\in {\mathbb{R}}^{B\times 1\times T\times F}. \end{array}$$ (4)

Here, ⊙ denotes the element-wise product. The absolute value is applied to ignore the sign of the gradient, retaining only the magnitude of the feature contribution.

##### 2.3.3.2 Band-level attribution aggregation

Clinical priors are defined on coarse-grained frequency bands. Therefore, we must aggregate the pixel-level saliency map **A** into a band-level probability distribution. First, we sum **A** along the time axis to obtain the cumulative saliency $\tilde{\text{A}}$ for each Mel frequency bin:


$$\begin{array}{l} \tilde{\text{A}}=\overset{T}{\underset{t=1}{\sum }}{\text{A}}_{:,:,t,:}\in {\mathbb{R}}^{B\times 1\times F}. \end{array}$$ (5)

We then normalize $\tilde{\text{A}}$ into a band attribution vector ${\text{p}}_{i}\in {\mathbb{R}}^{4}$ according to the four frequency band slices defined in the Section 2.3.1:


$$\begin{array}{l} p_{i,k}=\frac{\sum_{f\in {\text{band}}_{k}}{Ã}_{i,1,f}}{\sum_{j=1}^{4}\sum_{f\in {\text{band}}_{j}}{Ã}_{i,1,f}+\varepsilon },\;k\in \left\{1,2,3,4\right\}. \end{array}$$ (6)

Here, ε denotes a constant introduced for numerical stability, with a default value of 1 × 10^−8^. The vector **p**_*i*_ = [*p*_*i*, 1_, …, *p*_*i*, 4_] represents a probability distribution. It indicates the proportion of explanation weight the model assigns to the *k*-th frequency band.

##### 2.3.3.3 SCA loss and optimisation objective

For each sample *i*, we dynamically select the corresponding clinical prior distribution **π**_*i*_ based on its label *y*_*i*_:


$$\begin{array}{l} \pi_{i}=\{\begin{array}{ll} \pi^{\left(0\right)}, & y_{i}=0\;\left(\text{Normal}\right)\\ \pi^{\left(1\right)}, & y_{i}=1\;\left(\text{Abnormal}\right). \end{array} \end{array}$$ (7)

The core principle of SCA is to encourage the learnt attribution distribution **p**_*i*_ to align with the clinical prior **π**_*i*_. We use the KL divergence as the alignment metric. The SCA loss for a batch is defined as:


$$\begin{array}{l} L_{\text{SCA}}=\frac{1}{B}\overset{B}{\underset{i=1}{\sum }}D_{\text{KL}}\left({\text{p}}_{i}∥\pi_{i}\right)=\frac{1}{B}\overset{B}{\underset{i=1}{\sum }}\overset{4}{\underset{k=1}{\sum }}p_{i,k}\log\left(\frac{p_{i,k}}{\pi_{i,k}+\varepsilon }\right). \end{array}$$ (8)

The final training objective L comprises the standard cross-entropy loss L_CE_ and the SCA regularization term:


$$\begin{array}{l} L=L_{\text{CE}}+{\text{λ}}_{\text{sca}}\cdot L_{\text{SCA}}, \end{array}$$ (9)

where λ_sca_ is a hyperparameter balancing classification accuracy and prior alignment.

It is important to note that SCA introduces additional gradient computations only during the training phase. It does not alter the underlying model architecture or increase computational complexity during inference. Consequently, it serves as a lightweight, model-agnostic “clinical spectral aligner” that can be integrated with various backbone networks.

### 2.4 Model architectures

We utilize a 2D ResNet-18 network (He et al., 2016) as the universal feature extraction backbone. To accommodate single-channel log-Mel spectrograms, the initial convolutional layer (conv1) is modified to accept one input channel. We retain the original 7 × 7 kernel size, stride of 2, and padding of 3. The input tensor is represented as (*B*, 1, *T, F*), where *T* serves as the height (temporal dimension) and *F* as the width (frequency dimension). The final fully-connected layer is replaced with a linear layer containing two output units. This layer generates the 2D logits for “Normal” and “Abnormal” clinical outcomes.

**Figure 1:**
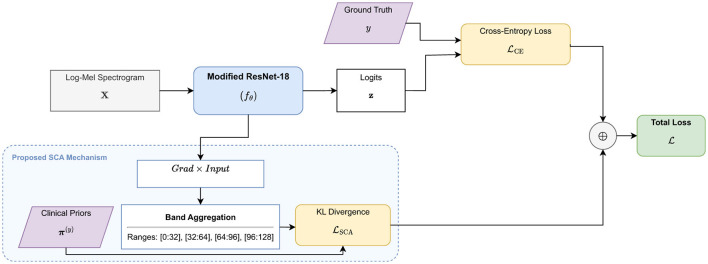
The SCA framework for heart sound classification. Log-Mel spectrograms **X** are processed by a modified ResNet-18 to generate clinical outcome logits. The model is optimized using a dual objective: cross-entropy loss L_CE_ for accuracy and SCA loss L_SCA_ for spectral alignment. SCA employs Gradient × Input to align band-level attributions with pre-defined clinical frequency priors **π**^(*y*)^ via KL divergence.

The architecture deliberately avoids complex multi-branch or multi-site fusion structures. This simplicity ensures that our analysis remains focused on the impact of clinical prior constraints on model performance and explainability. The baseline model is optimized using the standard cross-entropy loss L_CE_. We employ the Adam optimiser with a learning rate of 1 × 10^−3^ and a weight decay of 1 × 10^−4^. All model weights are randomly initialized. This protocol allows the training process to fully explore the feature extraction potential under spectral-guided SCA regularization. The workflow of our method is shown in Figure 1.

## 3 Experimental results

### 3.1 Classification performance

This section evaluates the classification performance of the proposed SCA framework in heart sound diagnosis. We adopt Accuracy, Sensitivity, and Specificity as core evaluation metrics. These provide a multi-dimensional perspective on diagnostic efficacy and misdiagnosis control, defined as follows:


$$\begin{array}{l} \text{ Acc}=\frac{TP+TN}{TP+TN+FP+FN},\\ \text{Sens}=\frac{TP}{TP+FN},\\ \text{Spec}=\frac{TN}{TN+FP}. \end{array}$$

where *TP*, *TN*, *FP*, and *FN* denote true positives, true negatives, false positives, and false negatives, respectively.

The experiments cover four distinct training protocols. CE-30 serves as the baseline using standard cross-entropy loss for 30 epochs. CE-60 extends this training to 60 epochs to observe the impact of increased duration. SCA-long employs a staged strategy. It starts with 30 epochs of standard training followed by 30 epochs of fine-tuning with the SCA regularization term. SCA-all utilizes a joint objective function containing the SCA loss for the full 60 epochs from random initialization.

Table 2 details the performance of these protocols on the PhysioNet 2016 and CirCor DigiScope datasets. To determine the optimal intensity of SCA regularization, we conducted a grid search on the validation set for λ_sca_ ∈ {0.05, 0.1, 0.2, 0.3, 0.5}. On the PhysioNet 2016 dataset, SCA-all attained an optimal accuracy of 94.14% at λ_sca_ = 0.30. The sensitivity reached 87.27%. This result is notably higher than the 78.18% achieved by CE-60. It suggests that SCA guides the model to capture pathologically significant features. This reduces the risk of missed diagnoses.

**Table 2:** Classification performance comparison across different training protocols. λ^*^ denotes the optimal regularization strength determined via grid search.

Protocol	λ^*^	PhysioNet 2016 (Val)			CirCor DigiScope (Test)		
		Acc (↑)	Sens (↑)	Spec (↑)	Acc (↑)	Sens (↑)	Spec (↑)
CE-30 (Baseline)	-	0.9290	0.8545	0.9442	0.5779	0.2628	0.8981
CE-60	-	0.9290	0.7818	**0.9591**	0.5595	0.2127	0.9118
SCA-long	0.05/0.10^†^	0.9259	0.8182	0.9480	**0.6044**	**0.2800**	0.9342
SCA-all	0.30/0.10^†^	**0.9414**	**0.8727**	0.9554	**0.6044**	0.2665	**0.9478**

^†^ Values indicate λ^*^ for PhysioNet 2016 and CirCor DigiScope, respectively. Bold values indicate the best performance in each dataset.

Experimental results on the more challenging CirCor DigiScope dataset demonstrate the robustness of SCA. Simply extending the training duration in CE-60 caused the test accuracy to decline from 57.79% to 55.95%. The model likely overfits to non-diagnostic acoustic artifacts such as environmental noise. Conversely, both SCA-all and SCA-long achieved the highest test accuracy of 60.44% at λ_sca_ = 0.10. SCA-all also exhibits a high specificity of 94.78%. This effectively suppresses false alarms in complex noise environments as excessive false positives caused by environmental artifacts can lead to alarm fatigue and decreased diagnostic trust among healthcare providers. A direct numerical comparison with existing literature remains challenging due to inconsistencies in training protocols, data partitioning, and pre-processing techniques across different studies. To the best of our knowledge, state-of-the-art performance on the PhysioNet 2016 dataset typically ranges between 91% and 97% accuracy (Er, 2021; Kaushal et al., 2023; Singh et al., 2024; Al-Tam et al., 2024), while reported accuracies on the CirCor DigiScope test set generally vary from 56% to 68% (Peng et al., 2022; Chang et al., 2025; Sabu et al., 2025; Gemke et al., 2022). Despite these variations, the SCA framework demonstrates strong competitiveness by achieving 94.14% and 60.44%, respectively, particularly as these results were obtained using a standard ResNet-18 backbone without complex architectural enhancements. These figures confirm that the proposed method reaches a performance level comparable to high-performing contemporary models. However, the primary target of our work extends beyond raw classification metrics. The core contribution lies in the framework's ability to align deep learning features with established clinical knowledge, a characteristic that is explored in detail through the explanability analysis in the subsequent section.

To ensure the reliability of our findings, we conducted a rigorous statistical analysis. We compared the baseline model (CE-60) against our optimal protocol (SCA-all). Table 3 presents the 95% confidence intervals (CIs) for all primary metrics. We computed these intervals using bootstrapping with 10,000 iterations. Additionally, we applied McNemar's test to evaluate the statistical significance of the predictions. For the challenging CirCor DigiScope dataset, SCA-all yields a highly significant improvement (*p* < 0.001). This confirms the robustness of our framework in noisy, real-world clinical environments. For the PhysioNet 2016 dataset, the accuracy and sensitivity demonstrate clear upward shifts within their CIs. However, the McNemar test does not reach statistical significance (*p* = 0.386). This non-significant result is a common statistical limitation. It stems from the extremely small validation set size (324 samples) combined with the ceiling effect of an already high baseline performance. Nevertheless, the consistent CI improvements across both datasets strongly validate the generalisability of the SCA methodology.

**Table 3:** Statistical validation comparing the CE-60 baseline and the SCA-all protocol. The 95% confidence intervals (in parentheses) are computed via bootstrapping (10,000 iterations). Statistical significance is determined using McNemar's test.

Dataset	Protocol	Accuracy (95% CI)	Sensitivity (95% CI)	Specificity (95% CI)	McNemar *p*-value
PhysioNet 2016	CE-60	0.9290 (0.8981–0.9568)	0.7818 (0.6667–0.8889)	**0.9591 (0.9338–0.9813)**	0.3865
	SCA-all	**0.9414 (0.9136–0.9660)**	**0.8727 (0.7778–0.9538)**	0.9554 (0.9288–0.9779)	
CirCor DigiScope	CE-60	0.5595 (0.5348–0.5835)	0.2127 (0.1848–0.2413)	0.9118 (0.8914–0.9310)	**< 0.001**
	SCA-all	**0.6044 (0.5804–0.6278)**	**0.2665 (0.2363–0.2968)**	**0.9478 (0.9323–0.9628)**	

Bold values indicate the best-performing protocol within each dataset.

### 3.2 Explainability analysis

#### 3.2.1 Quantitative analysis

To quantify the relationship between model attribution and frequency-domain priors, we employ KL divergence and Cosine similarity as the primary evaluation metrics. As shown in Table 4, the SCA-augmented models achieve superior alignment across both datasets. In the PhysioNet 2016 dataset, SCA-all considerably reduces the mean KL divergence to 0.1998 for normal samples and 0.3389 for abnormal samples. These results notably outperform the CE-60 baseline. Furthermore, the mean Cosine similarity for normal and abnormal recordings increases from 0.7718 and 0.7496 to 0.9013 and 0.7879, respectively.

**Table 4:** Quantitative explainability metrics comparing the CE-60 baseline and SCA protocols. Values are presented as (Normal/Abnormal).

Dataset/protocol	Mean KL Div (↓)	Mean Cosine Sim (↑)	*ΔKL*>0 Fraction (%)
PhysioNet 2016			
CE-60 (Baseline)	1.8526/3.5886	0.7718/0.7496	-
SCA-long	0.2058/0.4149	**0.9397/0.8217**	100.0/100.0
SCA-all	**0.1998/0.3389**	0.9013/0.7879	**99.6/100.0**
CirCor DigiScope			
CE-60 (Baseline)	0.7150/1.1689	0.8991/0.6907	-
SCA-long	0.4332/**0.2402**	0.7336/**0.8344**	59.9/99.8
SCA-all	**0.2872**/0.2676	0.8182/0.8336	**71.9/100.0**

Bold values indicate the best-performing of explainability metrics in each dataset.

The SCA framework notably enhances representational consistency across different signal categories. In the more challenging CirCor DigiScope dataset, the baseline model (CE-60) exhibits a sharp decline in Cosine similarity. Its performance drops from 0.8991 for normal samples to 0.6907 for abnormal recordings. This indicates a significant representational failure when encountering complex pathological murmurs. Conversely, SCA-all maintains stable and high-level alignment for both categories, with scores of 0.8182 and 0.8336. This cross-category robustness proves that SCA induces the model to learn universal clinical patterns. These patterns remain invariant to signal complexity. Despite the baseline having a higher Cosine similarity for normal samples in CirCor DigiScope, SCA-all achieves a far superior KL divergence of 0.2872. This confirms that SCA provides more precise probability distribution alignment by filtering out non-physiological artifacts.

**Figure 2:**
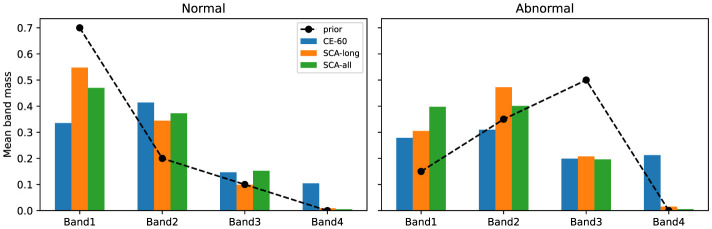
Mean band-level attribution mass for the PhysioNet 2016 dataset. For normal samples, attribution is concentrated in Band 1. For abnormal samples, the focus distinctly shifts toward Band 2 and Band 3, adhering to the frequency-informed prior.

**Figure 3:**
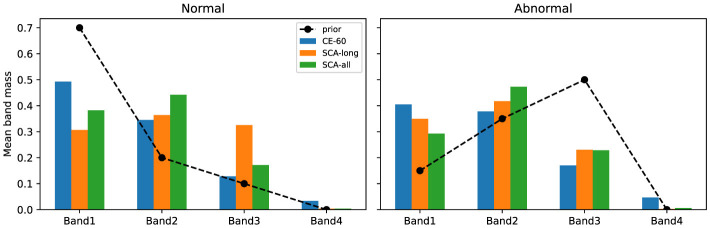
Mean band-level attribution mass for the CirCor DigiScope dataset. Despite environmental noise, the SCA framework maintains physiological alignment, ensuring that diagnostic focus remains within the clinically relevant frequency bands.

To assess the physiological plausibility of model decisions, we conducted a band-level attribution analysis on the validation set. The original attribution maps were calculated via the Grad × Input method. These maps were then aggregated into four Mel-bands based on clinically defined frequency ranges. Figures 2, 3 illustrate the mean weight distributions of these bands. In the PhysioNet 2016 dataset, SCA-all concentrates nearly 47% of the attribution weight in the first band (Band 1) for normal samples. This concentration aligns with the fundamental frequency ranges of S1 and S2 components. For abnormal samples, the attribution focus shifts significantly to Band 2 (approximately 40%) and Band 3 (approximately 20%). This shift effectively captures the high-frequency energy of pathological murmurs. In the CirCor DigiScope dataset, SCA-all maintains approximately 47% focus on Band 2 for abnormal samples despite environmental noise. Such active frequency-domain selection proves that the SCA framework learns robust physiological patterns.

**Figure 4:**
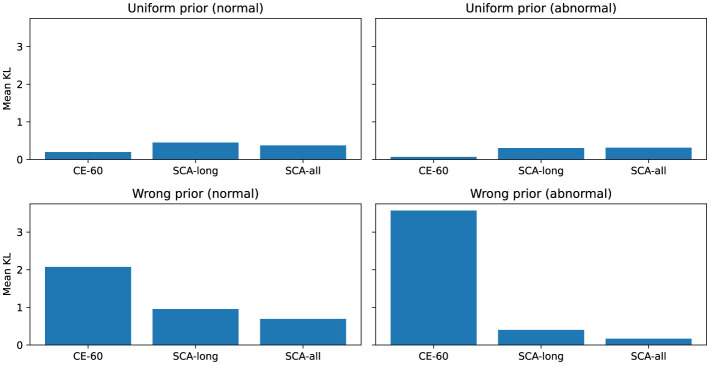
Sanity checks on PhysioNet 2016. The high KL divergence of the baseline model under the wrong prior highlights the fidelity of the SCA framework to correct clinical logic.

**Figure 5:**
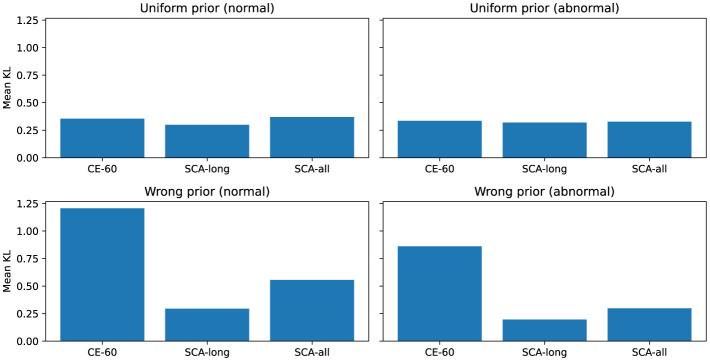
Sanity checks on CirCor DigiScope. These results confirm that the SCA gains are derived from specific clinical knowledge rather than a general reduction in feature entropy.

We introduced **Uniform** and **Wrong** priors as fidelity tests to verify feature alignment and exclude random interference (see Figures 4, 5). The Uniform prior represents an uninformed baseline where each band has an equal weight of 0.25. The Wrong prior simulates misleading instructions by deliberately inverting the importance of physiological frequency bands. Under the Wrong prior test for PhysioNet 2016 abnormal samples, the mean KL divergence for CE-60 increases sharply to approximately 3.6. This extreme value is driven by a support mismatch: CE-60 attributes predictions to the high-frequency noise band (Band 4). Since all defined priors assign near-zero probability to this noise zone, the baseline triggers a severe logarithmic penalty. Conversely, the SCA models successfully enforce noise suppression, thereby avoiding this penalty. While SCA-all exhibits a numerically low KL divergence (roughly 0.17) against the wrong abnormal prior, this indicates the successful avoidance of the noise-band penalty, not alignment with incorrect logic. As explicitly shown in the mean band mass distributions, SCA correctly shifts its attribution focus to the pathological mid-to-high frequency bands (Bands 2 and 3) for abnormal samples. It does not match the shape of the wrong prior, which assigns maximum weight to Band 1. Similarly, the KL divergence gap between protocols narrows under the Uniform prior in the CirCor DigiScope dataset. This analysis proves that SCA explicitly captures the correct clinical frequency-domain priors while rigorously enforcing noise suppression.

#### 3.2.2 Qualitative analysis

To verify the decision logic of the SCA framework, we performed qualitative visualization on representative samples using Grad × Input generates attribution maps as diffused, blurry clouds. These maps reflect the macro-level focus of the model on specific time-frequency regions. In contrast, the wavelet attribution method (WAM) (Kasmi et al., 2025) provides high spatio-temporal localization. It utilizes sharp patches or stripes to pinpoint specific acoustic events. This distinction in visual resolution directly reflects their fundamentally different roles within our methodology. Grad × Input is computationally efficient and produces smooth, continuous attribution distributions. This makes it mathematically well-suited for the coarse, band-level regularization employed during SCA training, ensuring stable end-to-end optimisation. In contrast, WAM involves computationally intensive multi-stage transformations to achieve its high-resolution localization. These discrete transformations make it inappropriate as a training-time optimisation signal, as it could disrupt gradient stability. Consequently, WAM is used to provide fine-grained visual evidence for clinical interpretation, while Grad × Input supports stable and effective optimisation under the proposed framework.

**Figure 6:**
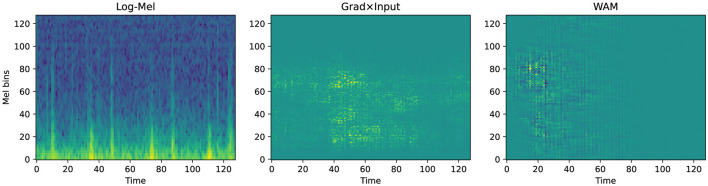
Qualitative attribution analysis for a normal sample from PhysioNet 2016 (idx = 313, *p* = 0.91).

**Figure 7:**
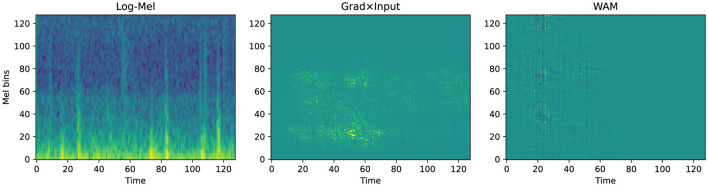
Qualitative attribution analysis for an abnormal sample from PhysioNet 2016 (idx = 126, *p* = 1).

In the normal case from the PhysioNet 2016 dataset (Figure 6), Grad × Input displays a diffused shadow in the low-frequency range between time steps 40 and 100. However, WAM identifies a strong reliance on the transient onset signal during the first 40 time steps. This attribution spans up to Mel bin 100, capturing the broad-frequency characteristics of the initial heart sound. Conversely, the abnormal sample in Figure 7 demonstrates a significant shift in the attribution focus. WAM highlights a cluster concentrated between Mel bins 40 and 100, particularly around time step 20. This focus aligns precisely with the high-frequency energy distribution of pathological murmurs.

**Figure 8:**
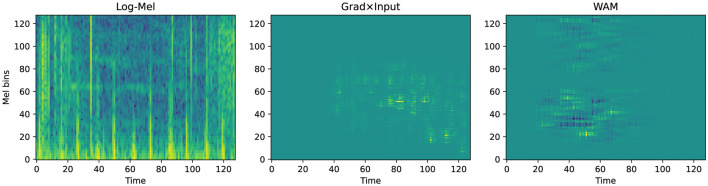
Qualitative attribution analysis for a normal sample from CirCor DigiScope (idx = 176, *p* = 0.60).

**Figure 9:**
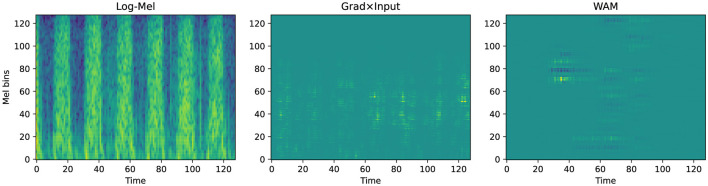
Qualitative attribution analysis for an abnormal sample from CirCor DigiScope (idx = 23, *p* = 0.96).

This physical alignment remains robust in the more complex CirCor DigiScope dataset. For the normal sample in Figure 8, WAM exhibits rhythmic attribution between Mel bins 20 and 60. The method effectively ignores the dense vertical background noise present in the log-Mel spectrogram. In the abnormal case shown in Figure 9, WAM identifies a highly localized pathological “hotspot” near Mel bin 80 at time step 40. This pinpointing avoids interference from low-frequency heart sound components.

These observations confirm that the SCA framework successfully induces clinical auscultation logic. The model identifies low-frequency physiological pulses in normal samples and captures mid-to-high frequency pathological murmurs in abnormal ones. The higher resolution provided by WAM confirms that this alignment is biologically plausible rather than a product of random noise.

**Figure 10:**
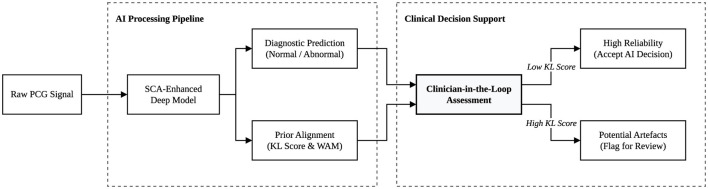
Schematic analysis of the SCA-enhanced clinical workflow. The diagram illustrates how the KL divergence serves as a clinical consistency score, enabling a clinician-in-the-loop process where AI predictions are cross-verified against established physiological priors and wavelet-based visual evidence.

In addition, to bridge the gap between quantitative alignment and clinical applicability, we interpret the KL divergence as a clinical consistency score. This score reflects the degree to which the model's decision is supported by physiologically plausible frequency regions. In practical deployment, this enables a clinician-in-the-loop workflow, as illustrated schematically in Figure 10. Predictions with low KL divergence can be considered more reliable and consistent with established auscultation principles. Conversely, predictions with high divergence may indicate reliance on non-diagnostic artifacts (e.g., environmental noise), thus flagging the need for further clinical verification. This mechanism transforms attribution alignment from a purely analytical metric into a clinically actionable signal for decision support.

### 3.3 Adaptability across architectures

To verify that the proposed SCA framework is model-agnostic, we conducted additional experiments using MobileNetV2 (Sandler et al., 2018) and DenseNet-121 (Huang et al., 2017). These models represent fundamentally different architectural paradigms compared to ResNet-18. MobileNetV2 utilizes depthwise separable convolutions. DenseNet-121 employs dense feature block connectivity. We evaluated these backbones on the PhysioNet 2016 dataset.

As shown in Table 5, integrating the SCA regularization yields consistent improvements in explainability across all architectures. For MobileNetV2, the SCA-all protocol drastically reduces the mean KL divergence for normal samples from 3.2259 to 0.1415. Similar substantial reductions occur in DenseNet-121. While there is a marginal decrease in overall accuracy (< 1.0%), this represents a well-documented accuracy-interpretability trade-off. The models sacrifice negligible performance to achieve strict physiological alignment. This cross-architecture validation confirms that SCA functions as a universal, plug-and-play spectral aligner. It consistently enhances physiological plausibility across diverse deep learning backbones.

**Table 5:** Performance and explainability metrics across diverse backbone architectures on the PhysioNet 2016 dataset.

Backbone	Protocol	Accuracy (↑)	Mean KL (normal) (↓)	Mean KL (abnormal) (↓)
ResNet-18	CE-60 (Baseline)	0.9290	1.8526	3.5886
	SCA-all	**0.9414**	**0.1998**	**0.3389**
MobileNetV2	CE-60 (Baseline)	**0.9259**	3.2259	1.9781
	SCA-all	0.9167	**0.1415**	**0.6159**
DenseNet-121	CE-60 (Baseline)	**0.9290**	1.6131	3.1850
	SCA-all	0.9259	**0.2787**	**0.6785**

Bold values indicate the best-performing protocol within each backbone architecture.

### 3.4 Ablation study

To investigate the specific sources of performance gain, we conducted ablation experiments on the PhysioNet 2016 and CirCor DigiScope datasets. These ablation analyses are confined entirely to the validation sets to prevent data leakage and preserve the integrity of blind testing.

Table 6 presents the results of the band masking analysis. We applied hard masking to the input Mel spectrograms to evaluate the reliance of the models on specific physiological frequency bands. Frequency ranges were divided into low-band (Band 1+2) and high-band (Band 3+4) regions. In the PhysioNet 2016 dataset, restricting the input to Band 1+2 caused the sensitivity of the CE-60 baseline to drop to 16.36%. In contrast, SCA-all maintained a sensitivity of 78.18% and an accuracy of 93.21% under the same conditions. This demonstrates that the SCA framework significantly strengthens the model's ability to capture core physiological features, such as S1 and S2 components. In the CirCor DigiScope dataset, SCA-all achieved a sensitivity of 47.54% under low-band masking, which is higher than the 34.43% achieved by the baseline. This result verifies the spectral selection advantage of SCA in noisy environments. When restricted to the high-band (Band 3+4), the sensitivity of all models approached zero. This supports the clinical consensus that heart sound diagnosis must rely on fundamental physiological pulses.

**Table 6:** Cross-dataset band masking analysis results on the validation sets.

Dataset	Protocol	Masking mode	Acc (↑)	Sens (↑)	Spec (↑)
PhysioNet 2016	CE-60 (baseline)	None	0.9290	0.7818	0.9591
	SCA-all	None	**0.9414**	**0.8727**	0.9554
	CE-60 (Baseline)	Low-band	0.8549	0.1636	**0.9963**
	SCA-all	Low-band	0.9321	**0.7818**	0.9628
CirCor DigiScope	CE-60 (baseline)	None	0.6872	0.3060	0.9175
	SCA-all	None	0.7181	0.3060	**0.9670**
	CE-60 (baseline)	Low-band	**0.6934**	0.3443	0.9043
	SCA-all	Low-band	0.6728	**0.4754**	0.7921

Bold values indicate the best-performing protocol under each masking condition within each dataset

Furthermore, we compare SCA with *L*_2_ regularization to determine if the performance gains are merely due to general feature constraints. We apply an *L*_2_ penalty to the penultimate features, denoted by *h*, which represent the global latent representations extracted by the CNN backbone prior to the final fully connected layer (*CE* + λ||*h*||^2^). We perform a grid search for λ ∈ {0.05, 0.1, 0.2, 0.3, 0.5} during a 60-epoch training process. Table 7 summarizes these results. In the PhysioNet 2016 dataset, SCA-all outperforms the best-performing *L*_2_ configuration in both accuracy (94.14%) and sensitivity (87.27%). In the CirCor DigiScope validation set, *L*_2_ regularization achieves a slightly higher accuracy of 72.22%. However, SCA-all provides superior specificity. Minimizing false positives is essential in clinical practice to prevent alarm fatigue. Unlike the uninformed penalty of the *L*_2_ norm, SCA provides *informed regularization* by explicitly capturing clinical priors. This approach ensures that performance gains stem from biologically plausible patterns rather than arbitrary entropy reduction.

**Table 7:** Comparison of feature regularization and SCA protocols on the validation sets.

Dataset	Protocol	Parameter (λ_*L*_2__)	Acc (↑)	Sens (↑)	Spec (↑)
PhysioNet 2016	CE-60 (baseline)	–	0.9290	0.7818	0.9591
	*L*_2_ Regularization	0.30	0.9383	0.8000	**0.9665**
	SCA-all	–	**0.9414**	**0.8727**	0.9554
CirCor DigiScope	CE-60 (baseline)	–	0.6872	0.3060	0.9175
	*L*_2_ regularization	0.10	**0.7222**	**0.3607**	0.9406
	SCA-all	–	0.7181	0.3060	**0.9670**

Bold values indicate the best-performing protocol within each dataset.

## 4 Discussion and conclusion

In this study, we proposed and systematically evaluated the Scale-Consistent Attribution (SCA) framework for explainable prior alignment in heart sound screening. Methodologically, we partitioned the log-Mel spectrogram into four clinically relevant frequency bands. We encoded the domain knowledge into soft priors. During the training phase, we applied KL regularization to the band-level attribution distributions aggregated via Grad × Input to explicitly constrain the model's focus. On the PhysioNet 2016 and CirCor DigiScope benchmarks, we observed that SCA achieved classification performance comparable to or slightly better than the baseline. However, SCA considerably reduced the KL distance between the attribution distribution and clinical priors, particularly in abnormal samples. This gap narrowed under uninformed uniform priors, while the KL patterns reversed under deliberately constructed “wrong priors.” Fidelity tests and ablation experiments further confirmed that these performance gains did not originate from general feature entropy reduction. Instead, through “informed regularization,” the model learnt biologically plausible discriminative patterns by locking onto low-frequency physiological pulses in normal samples and capturing mid-to-high frequency pathological energy in abnormal cases.

This research also possesses several limitations. First, the framework uses unified and coarse band-level soft priors. It does not explicitly incorporate fine-grained physiological information, such as S1/S2 segmentation, systolic/diastolic phases, or specific murmur types. Therefore, SCA currently serves as a first-step frequency-domain constraint rather than a complete model of heart sound mechanisms. Second, the explainability assessment relies primarily on Grad × Input band-level metrics. Time-frequency visualizations like WAM serve only as qualitative case studies. A systematic comparison of multiple methods and subjective clinician evaluations are still lacking. It also remains unverified whether superior prior alignment directly improves clinical usability. Finally, the method is validated only on the ResNet18 architecture and two public dataset splits. It has not undergone comprehensive comparison with top-performing challenge models or multi-task hierarchical modeling.

To address these limitations and mitigate real-world clinical risks, future work will extend the SCA framework. We will focus on adaptive and context-aware prior design. Device-specific artifacts, such as non-stationary stethoscope friction or environmental noise, require targeted solutions. Dynamic priors could continuously estimate and down-weight these noise-dominated frequency regions. Similarly, rare cardiovascular pathologies often exhibit non-standard spectral characteristics. The framework can accommodate these anomalies through flexible or disease-specific priors, alongside finer phase-band modeling. These adaptations aim to preserve the core principle of physiological plausibility. They also improve model robustness under diverse and uncertain clinical conditions. Crucially, the KL divergence will retain its role as a clinical consistency score. This metric provides a clear signal to identify potentially unreliable predictions. Consequently, it strongly supports risk-aware clinical interpretation. Ultimately, these methodological advancements must integrate comprehensive expert clinical evaluations. This step is essential to move beyond mere prior alignment. It ensures safer and more effective AI-assisted decision-making.

## Data Availability

The PhysioNet 2016 dataset used in this study is publicly available at: https://physionet.org/content/challenge-2016/1.0.0/. The CirCor DigiScope dataset is publicly available at: https://physionet.org/content/circor-heart-sound/1.0.3/. The code for this study is available at: https://github.com/glam-imperial/heartsound_SCA.
